# Integrative genomic analysis identifies key target genes and candidate drugs for spinal stenosis

**DOI:** 10.3389/fnmol.2026.1767263

**Published:** 2026-04-01

**Authors:** Demeng Xia, Yongjie Chen, Rui Wu, Yifan Tang, Yanqing Sun, Xiongsheng Chen

**Affiliations:** 1 Department of Orthopedics, Shanghai General Hospital, Shanghai Jiao Tong University School of Medicine, Shanghai, China; 2 Key Laboratory of Carcinogenesis and Translational Research (Ministry of Education), Department of Thoracic Surgery II, Peking University Cancer Hospital and Institute, Beijing, China

**Keywords:** molecular docking, single-cell RNA sequencing, spinal stenosis, summary-data-based Mendelian randomization, target genes

## Abstract

**Background:**

Spinal stenosis is a common pathological condition characterized by the narrowing of the spinal canal, contributing to substantial morbidity and imposing a significant socioeconomic burden. Despite its clinical importance, the genetic drivers and cellular mechanisms driving its progression remain inadequately understood, necessitating integrative approaches to identify therapeutic targets.

**Methods:**

This study employed an integrative multi-omics strategy. Initially, summary-data-based Mendelian randomization was conducted using cis-expression quantitative trait loci data from 19,960 genes alongside spinal stenosis genome-wide association study data. Gene-gene interaction networks and colocalization analyses further refined candidate genes. Additionally, single-cell RNA sequencing of spinal tissues was performed to assess cellular enrichment, and molecular docking was employed to screened FDA-approved drugs against prioritized targets. Immunohistochemistry (IHC), Western blot (WB), and quantitative real-time PCR (qRT-PCR) were conducted using tissue samples and primary cells to validate the bioinformatics analysis results.

**Results:**

SMR analysis identified 45 candidate target genes, which were further narrowed to three key genes including KAT5, TET2, and TAF10 through gene-gene interaction analysis and colocalization. Single-cell RNA sequencing revealed that these genes were predominantly enriched in chondrocytes and monocytes, implicating cellular cross-talk via the TGF-β1– (TGF-βR1 ++ TGF-βR2) pathway, a driver of fibrosis and ossification. Molecular docking identified six high-affinity compounds: Balsalazide and Eltrombopag for KAT5, Magnesium Citrate and Ferric Citrate for TET2, and Piracetam and Deferiprone for TAF10. The expression level of KAT5 and TET10 were both consistent with our SMR analysis in both tissues and primary cells.

**Conclusion:**

These findings elucidate novel genetic and cellular mechanisms underlying spinal stenosis, highlighting the role of TGF-β pathway in disease progression. The identified compounds offer promising therapeutic interventions, bridging genomic discoveries to clinical applications and paving the way for targeted treatment strategies.

## Graphical Abstract

**Figure:**
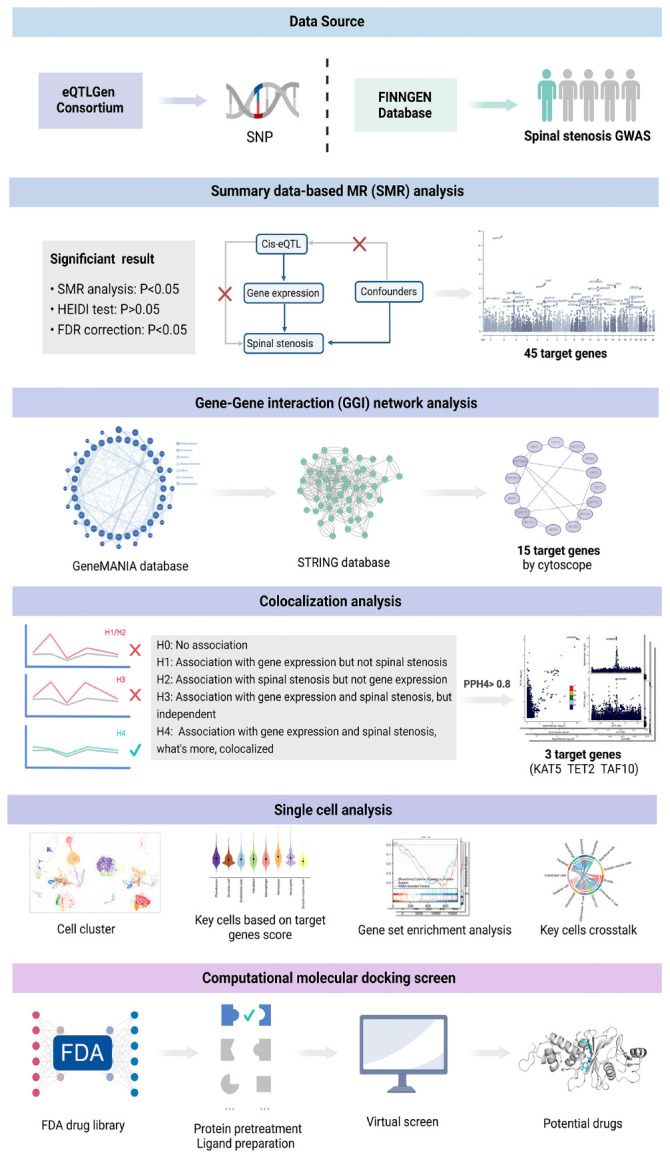
The flowchart of our study. The target genes were step-by-step selected by SMR analysis, GGI network analysis and colocazation analysis. Single cell analysis verified the target genes and enriched the target pathway. Computational molecular docking screen predicted the potential therapeutic drugs.

## Introduction

Spinal stenosis is a pathological condition characterized by the narrowing of the spinal canal, which can lead to compression of the spinal cord or nerve roots (Katz et al., 2022). This narrowing typically occurs in the neck or lumbar regions, though it can occur throughout the spine. It is estimated to affect approximately 11% of the general population, particularly the elderly (Jensen et al., 2021). Globally, around 103 million individuals experience symptoms of lumbar spinal stenosis, and in the United States alone, over 350,000 individuals aged 45 years or older undergo decompressive laminectomy annually, while an additional 370,000 undergo lumbar fusion surgery (Kreiner et al., 2013). The clinical presentation of spinal stenosis varies depending on the location and severity of the narrowing (McIlroy et al., 2021). Common symptoms include pain, numbness, weakness, and functional impairment (Wang et al., 2024). When the condition affects the cervical spine, symptoms may include neck pain, radicular arm pain, and in severe cases, myelopathy, which can manifest as hand clumsiness, gait disturbances, and even bowel or bladder dysfunction (Weiner et al., 2021). Some of patients could also developed neurogenic claudication which takes pain, cramping, or weakness in the lower extremities as main symptoms, typically exacerbated by walking or standing and relieved by sitting or bending forward. Another phenomenon is that some individuals may remain asymptomatic, while others may experience significant disability even with the same severity of the narrowing (Chen et al., 2024). Given that severe spinal stenosis can lead to paralysis and imposes a substantial socioeconomic burden, it has drawn increasing research attention (Jensen et al., 2021).

Spinal stenosis may arise from degenerative changes, congenital abnormalities, trauma, spinal disorders, and inflammatory conditions (Zhu et al., 2024). It is most commonly associated with age-related degeneration, including osteoarthritis, disc degeneration, and ligament thickening, which progressively reduce spinal canal space, particularly in individuals over 50 years of age (Lai et al., 2020). Degenerative changes lead to central canal, lateral recess, or intervertebral foraminal narrowing, often accompanied by facet joint arthrosis and ligamentum flavum hypertrophy (Zaina et al., 2016). Such narrowing may compress neural and vascular structures, resulting in ischemia and neurological symptoms. Additionally, individuals with a congenitally narrow spinal canal are predisposed to stenosis, particularly when compounded by degenerative processes in later life (Le Huec et al., 2016). Furthermore, conditions such as scoliosis, spondylolisthesis, and spinal tumors can contribute to stenosis by altering spinal alignment and integrity, while inflammatory diseases like ankylosing spondylitis and rheumatoid arthritis may induce inflammatory and structural changes, further exacerbating spinal stenosis (Bussières et al., 2021).

As an idiopathic multifactorial disease, spinal stenosis is influenced by both genetic and postnatal factors. Evidence suggests that specific single nucleotide polymorphisms (SNPs) contribute to spinal canal abnormalities, including ossification of the posterior longitudinal ligament (OPLL) and congenital stenosis, thereby increasing susceptibility to spinal stenosis (Fan et al., 2024). These genes may promote phenotypic changes in spinal cells, directly leading to structural abnormalities, and modulating their regulation could present novel therapeutic opportunities (Clos et al., 2021; Pruna et al., 2013). However, the precise relationship between SNPs and spinal stenosis remains unclear, and their mechanisms are not yet fully elucidated, necessitating further systematic genetic research (Aicale et al., 2019; Pruna et al., 2017). Therefore, we applied summary-data-based Mendelian randomization (SMR) and colocalization analysis to investigate causal associations between SNPs and spinal stenosis, while gene-gene interaction (GGI) analysis was performed to identify key genes (Oh et al., 2024). To further explore the role of these genes, single-cell RNA sequencing (SCS) analysis of spinal tissues was conducted. SCS enables a more precise characterization of cellular heterogeneity and biological complexity in spinal stenosis at an unprecedented resolution (Mahat et al., 2024). Based on these findings, molecular docking analysis was employed to identify potential therapeutic drugs, offering insights into targeted interventions for spinal stenosis treatment. Finally, we performed immunohistochemistry staining of target proteins on posterior longitudinal ligament tissues and Western Blot on primary cells digested by trypsin from posterior longitudinal ligament (PLL) tissues of patients with OPLL and PLL tissues of health people to validate our analysis results.

## Materials and methods

### Study design

The flowchart of this research was illustrated in Graph Abstract. This research comprises multiple analytical components, including summary-data-based Mendelian randomization (SMR) analysis, protein-protein interaction network analysis, single-cell RNA sequencing (SCS) analysis of spinal stenosis samples, and computational molecular docking for drug screening. We initially performed SMR analysis to identify genes with causal associations with spinal stenosis risk and further refined key genes using gene-gene interaction (GGI) and colocalization analysis. SCS analysis was conducted to examine key gene expression patterns across different cell subsets within spinal stenosis samples, validating previous analyses and identifying monocyte-chondrocyte interactions as a potential pathogenic mechanism. Finally, molecular docking was applied to screen FDA-approved drugs targeting the identified key genes, predicting potential therapeutic candidates.

### MR analysis

#### Data sources

Gene expression data for 19,960 genes was obtained from the expression quantitative trait loci (eQTL) Gen Consortium, which aggregates 37 datasets from 31,684 individuals, first published by Dutch researchers in 2021 (Võsa et al., 2021). The link was^1^ eQTL can be classified into cis-eQTL and trans-eQTL, where cis-eQTL mostly affect the expression levels of genes by a gene-proximal (<1 Mb) SNP, the SNP in trans-eQTL locates distal to the gene (>5 Mb), even on a different chromosome. Only Cis-eQTL information was included in this study. As eQTL has become an important tool to interpret regulatory mechanisms of variants identified by genome-wide association studies (GWASs), combined with spinal stenosis GWAS database, this one of the largest eQTL database which could help us explore the heritability of certain disease by play the role of exposure. Spinal stenosis acts as the outcome in this MR analysis. The SNP information of spinal stenosis was acquired from FinnGen Database Version R10 according to https://www.finngen.fi/en, which included 20,807 spinal stenosis samples and 294,770 normal samples, 21,311,942 variants and 2,408 endpoints released on December 2023.

#### SMR analyses, gene-gene interaction network analysis and colocalization study

We conducted summary-data-based Mendelian randomization (SMR) using 19,960 genes as exposures, spinal stenosis as the outcome, and eQTLs as instrumental variables (IVs). Genes exhibiting significant differential expression (DEGs) were considered key candidates. IVs were selected using genome-wide significance thresholds (*p* < 5 × 10^−8^) to ensure robust associations with the exposure. Clumping procedures (clump = TRUE) were applied to minimize linkage disequilibrium (LD) effects, employing strict parameters (LD threshold *r*^2^ = 0.001, genomic window = 10 kb) to exclude variants with allelic correlation (Huang et al., 2021).

To distinguish true pleiotropy from LD-related confounding, we applied the heterogeneity in dependent instruments (HEIDI) test alongside SMR, ensuring robustness against confounding from neighboring genetic variants using a criterion of *p* > 0.05 (Zhu et al., 2016). False discovery rate (FDR) adjust was also included to improve the accuracy of hypothesis testing (*p* < 0.05). By ranking *p*-value according to its numeric value, this algorithm adjusts p into q, q = p*n/rank.

Among 19,960 genes, only 45 passed the MR significance thresholds. To further validate and prioritize functionally relevant genes, we incorporated GeneMANIA, an online tool integrating genomic, transcriptomic, and proteomic data, to predict gene functions and discover novel functional associations (Franz et al., 2018). Protein-protein interaction (PPI) analysis was conducted using the STRING database, followed by further filtering via Cytoscape, ultimately narrowing the 15 most crucial hub genes.

Colocalization analysis, which complements SMR, was employed to determine whether the same genetic variant influences both gene expression and spinal stenosis risk, strengthening causal inference (Wu S. et al., 2024). This approach assesses the probability that two traits share a common causal variant within a genomic region, thereby reducing false positives caused by LD or coincidental signal overlap of association signals. Using a posterior probability (PPH4) threshold > 0.8, three target genes were finally acquired.

#### Single-cell sequencing SCS data analysis and cell interaction analysis

SCS data was acquired from GEO website, project GSE271018 and Seurat R package (v4.3.0) was applied in data preprocessing, dimensionality reduction, cluster analysis and differential gene identification (Tang et al., 2024). The link of project GSE271018 is https://www.ncbi.nlm.nih.gov/geo/query/acc.cgi?acc=GSE271018. Cells with < 200 genes and genes appearing in < 5 cells were both excluded. The number of nfeature_RNA was set as 200–4,000, the number of nCounts was set as < 2,000 and the percent.mt was set as < 30%. Following standardization, 2,000 highly variable genes were selected. Principal component analysis (PCA) was employed for dimensionality reduction, while UMAP was used for visualization. Top 10 principal components (PCs) were selected based on variance distribution (dims = 10). Cell clustering was performed using a resolution of 0.5, and cell types were manually annotated based on marker gene identification and literature-based validation.

To evaluate the coordinated transcriptional activity of the three causally supported genes (KAT5, TET2, and TAF10), we constructed a user-defined gene set (termed a “virtual pathway”) and applied the AddModuleScore function in Seurat (v4.3.0). Module scores were calculated by averaging the expression of the target genes in each cell and subtracting expression-matched background gene sets to minimize technical biases. The average module score across cell clusters was used to determine cell-type enrichment. The chondrocyte and monocyte were picked out as the highest expression level of target genes. The single-gene pathway enrichment analyses were also performed by Gene Set Enrichment Analysis (GSEA). Significantly enriched pathways were identified using the hypergeometric test based on the C2 canonical pathway gene set (MSigDB, v2022.1) and filtered by statistical significance (*p* < 0.05) and false discovery rate (FDR < 0.25).

To investigate cell-cell interactions, we employed the CellChat R package (CellChat v1.6.1), which quantifies ligand-receptor pair interactions using the CellChatDB database. This analysis translates gene expression into intercellular communication dynamics, facilitating insights into monocyte-chondrocyte cross-talk in spinal stenosis pathogenesis.

#### Molecular docking and potential targeting drugs prediction

The three-dimensional (3D) structures of active compounds were retrieved from the FDA Launched Drug Library. Compounds were optimized using ChemBio3D 14.0, which adjusted spatial conformation and energy minimization, saving files in mol2 format. Preprocessing in AutoDockTools 1.5.6 converted the files into pdbq format (Morris et al., 2009). The 3D crystal structures of target proteins were downloaded from UniProt. Water molecules and non-relevant organic matter were removed using Notepad2, followed by hydrogenation, charge distribution, and atomic type addition using AutoDockTools 1.5.6, with final structures saved in pdbqt format (Trott and Olson, 2010). Molecular docking was executed using AutoDockVina, and docking results were visualized with PyMOL 2.6 (Eberhardt et al., 2021).

#### Primary cells extraction, immunohistochemistry staining, and Western blot validation

After being fixed, deparaffinized, and hydrated, we managed to acquire both ligament tissues of OPLL and PLL in intact form from Department of Orthopedics, Shanghai General Hospital by professional spine surgeons between July 2025 and November 2025. The study was approved by the Ethics Committee of Shanghai General Hospital and was conducted in accordance with the Declaration of Helsinki. The pathology type of each patient was examined by two experienced pathologists and two experienced spine surgeons. Posterior longitudinal ligament tissues were collected from 3 patients diagnosed with OPLL and 3 control patients without ossification undergoing spinal surgery. OPLL represents a major pathological contributor to spinal canal narrowing characterized by fibrosis and ectopic ossification, and was therefore selected as a clinically relevant validation model.

Then antigens were retrieved by boiling tissues with sodium citrate buffer (10 mM, pH 6.0) in pressure cocker for 10 min. After washed by PBS, the slides were blocked by 10% goat serum for 30 min at room temperature, relatively labeled overnight at 4°C with an anti-KAT5 antibody (Abcam, Cambridge, United Kingdom) and an anti-TET2 antibody (Abcam, Cambridge, United Kingdom) and the secondary antibody was later added and incubated for 40 min at room temperature. Finally, tissues were stained with 1 × DAB solution and hematoxylin. The image was taken by optical microscope at 200× magnification and the total staining score was calculated as the product of the staining-intensity score and the staining-positivity rate.

By using 0.25% trypsin, we managed to separate primary cells from OPLL and PLL tissues (*N* = 3) and culture them in DMEM medium with 10% FBS and 1 × Penicillin-Streptomycin Solution in 10 cm dishes. After removing the culture medium, 2 mL PBS was used to wash the cells and repeated it three times. After the clean, 1 × RIPA lysate was added and ultrasonic cell disruptor was applied to extract proteins from cells. BCA assay kit was used to detect protein concentration. The products were separated by SDS-page and transferred to PVDF membranes. After blocking the membranes with 5% BSA diluted by TBST on shaker for 1 h at room temperature, they were relatively incubated with the indicated primary antibodies including KAT5 and TET2 at 4°C overnight and then were incubated with the GADPH antibody. Then they were incubated with horseradish peroxidase-conjugated secondary rabbit antibodies at room temperature for 1 h. Amersham Imager 600 was used to detect chemiluminescence signals.

## Results

### SMR analysis reveals 45 potential target genes

**FIGURE 1:**
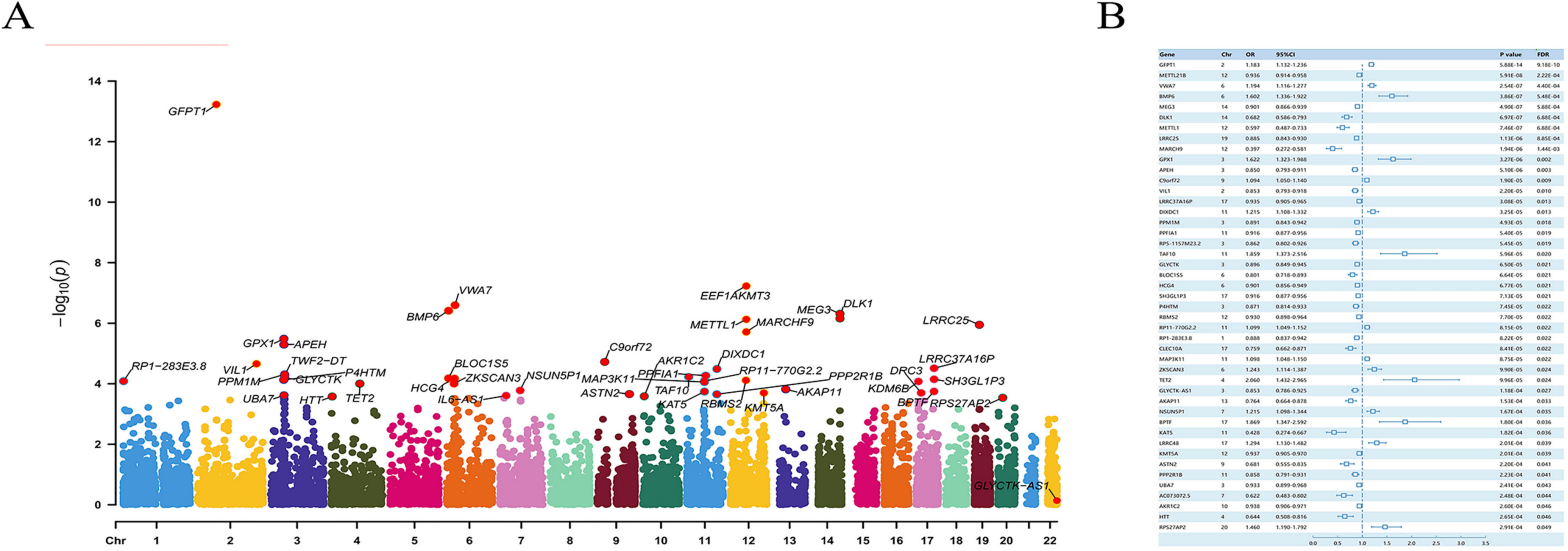
The SMR analysis results. **(A)** The Manhattan plot of genes’ correlation significance. **(B)** The detailed information of the correlation between selected 45 genes and stenosis shown in forest map.

To select genes which can induce or prevent the occurrence of spinal stenosis, 19,960 genes were included in the SMR analysis and only 45 of them were picked out as having causal relationship with spinal stenosis according to the standard of SMR analysis *p* < 0.05, HEIDI test *p* > 0.05 and FDR correction *p* < 0.05. 15 of those potential target genes including GFPT1, VWA7, BMP6, GPX1, C9orf72, DIXDC1, TAF10, RP11-770G2.2, MAP3K11, ZKSCAN3, TET2, NSUN5P1, BPTF, LRRC48, and RPS27AP2, have positive correlation with spinal stenosis while the other 30 genes including METTL21B, MEG3, DLK1, METTL1, LRRC25, MARCH9, APEH, VIL1, LRRC37A16P, PPM1M, PPFIA1, RPS-1157M23.2, GLYCTK, BLOC1S5, HCG4, SH3GL1P3, P4HTM, RBMS2, RP1-283E3.8, CLEC10A, GLYCTK-AS1, AKAP11, KAT5, KMT5A, ASTN2, PPP2R1B, UBA7, AC073073.5, AKR1C2, and HTT, have negative correlation with spinal stenosis. Of 45 candidate genes, about 73% (33/45) were negatively correlated with stenosis, suggesting protective roles. The significance of the correlation between each gene and spinal stenosis were showed in Figure 1A and the correlating relationship between 45 genes and spinal stenosis were shown in Figure 1B.

### GGI network analysis picked out 15 hub genes

**FIGURE 2:**
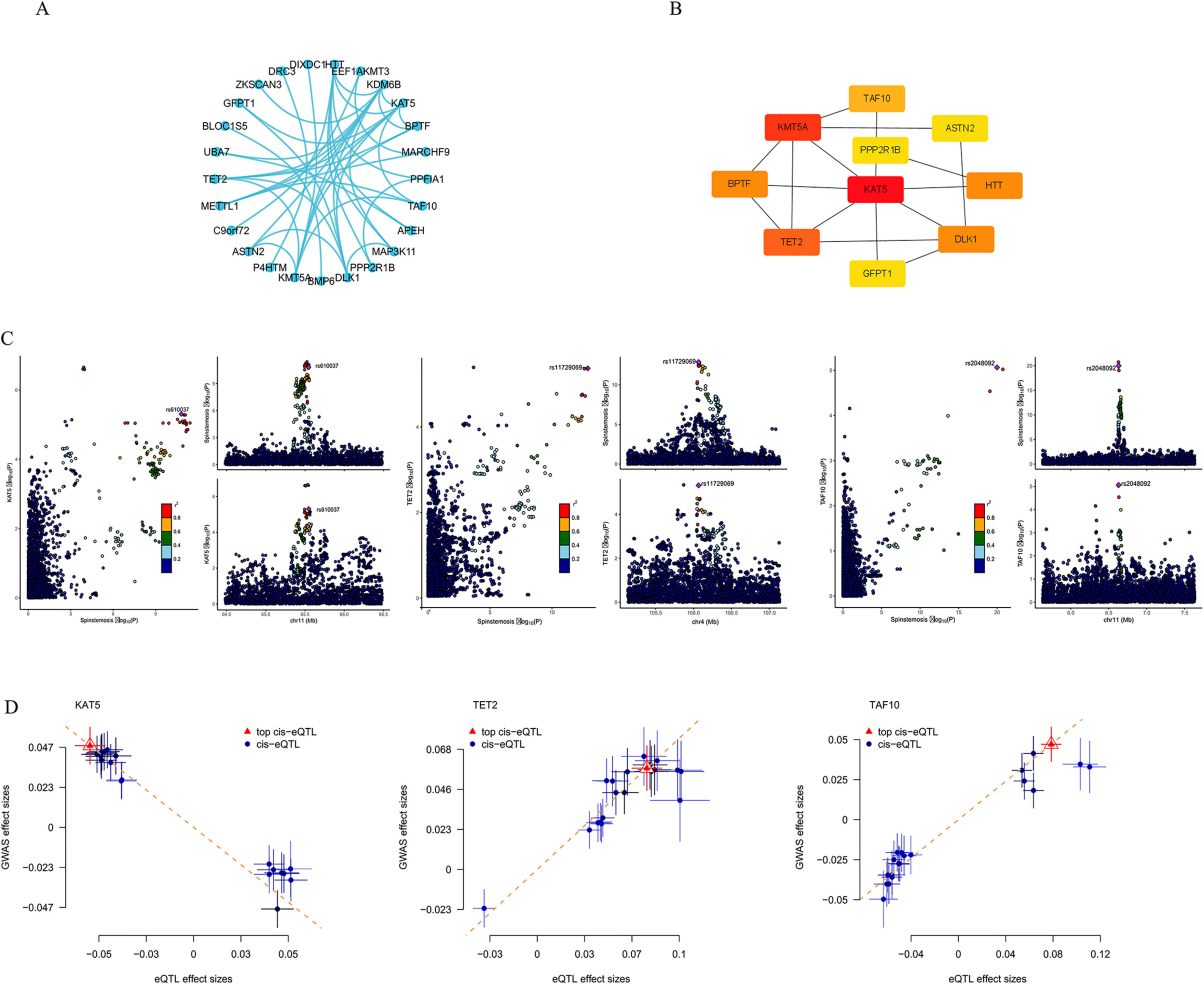
The PPI network analysis results and colocalization analysis results. **(A)** The PPI network built on String database. **(B)** The string graph built by Cytoscape software. **(C)** The colocalization analysis results of the three target genes. **(D)** The correlation between three target genes and spinal stenosis shown in scatter plot.

The interaction between genes and genes and the interaction between proteins and proteins were both applied to further select the potential target genes from 45 genes. The GGI network was built by GeneMANIA platform (Figure 2A) and the PPI network was built on string database (Figure 2B). After the selection based on these two networks, we picked out 15 hub genes including TAF10, AKR1C2, APEH, KAT5, TET2, GFPT2, GGFIA1, SETD8, WDR4, METTL1, GFPT1, BPTF, PPP2R1B, PPP2R5D, and MAZ (Figure 2C). KAT5, GFPT2, GFPT1, and PPP2R5D seems being the four genes with the strongest interaction.

### Colocalization analysis screened three target genes

We managed to quantified the genetic influences of 15 hub genes on spinal stenosis. Only three genes, KAT5, TET2, and TAF10 passed through colocalization analysis (Figure 2C). While KAT5 (OR, 0.428; 95% CI 0.274–0.667; FDR = 0.036) has negative correlation with spinal stenosis, both TET2 (OR, 2.060; 95% CI 1.432–2.965; FDR = 0.024) and TAF10 (OR, 1.859; 95% CI 1.373–2.516; FDR = 0.020) have positive correlation with spinal stenosis (Figure 2D).

### Single cell analysis

**FIGURE 3:**
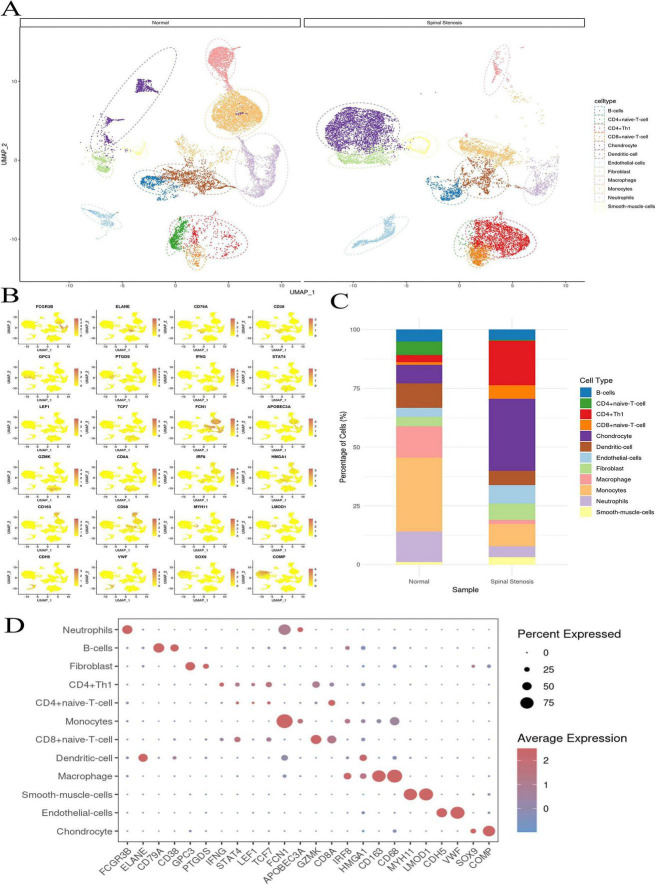
Primary SCS analysis results. **(A)** The cell type distribution in normal tissue and spinal stenosis tissue. **(B)** The marker distribution of cell type in tissues. **(C)** The percentage contrast of cell types in normal tissues and spinal tissues. **(D)** The markers composition of different cell types.

SCS analysis was conducted on database GSE271018 which included bone and ligament tissues from four patients with degenerative lumbar stenosis and two patients without the condition. A totally of 43,747 cells were classified into 12 cell clusters, including B cells, CD4+ naive T cell, CD4+ Th1, CD8+ naive T cell, chondrocyte, dendritic cell, endothelia-cells, fibroblast, macrophage, monocytes, neutrophils and smooth-muscle-cells (Figure 3A). The distribution situation of cell-type-specific markers was shown in Figure 3B. Comparision of cell type proportions between normal sample and spinal stenosis sample shows that the CD4+ naive T cell witnessed a clear decrease while both CD4+ Th1 and CD8+ naive T cell both witnessed significant increase (Figure 3C), suggesting a potential association between the occurrence of spinal stenosis and aseptic inflammatory responses. Additionally, chondrocyte and smooth-muscle cells exhibited a notable upward trend which were consistent with OPLL and other factors that directly lead to spinal stenosis. The expression levels of 24 markers in all 12 cell types were shown in Figure 3D. TCF7 is a specific marker for monocytes and COMP is exclusively expressed in chondrocytes. In conjunction with TCF7, FCGR3B serves as a reliable marker for neutrophils. Macrophages, smooth-muscle-cells and endothelial-cells were all marked by at least two genes as single marker could not meet the criteria of distinguishment.

**FIGURE 4:**
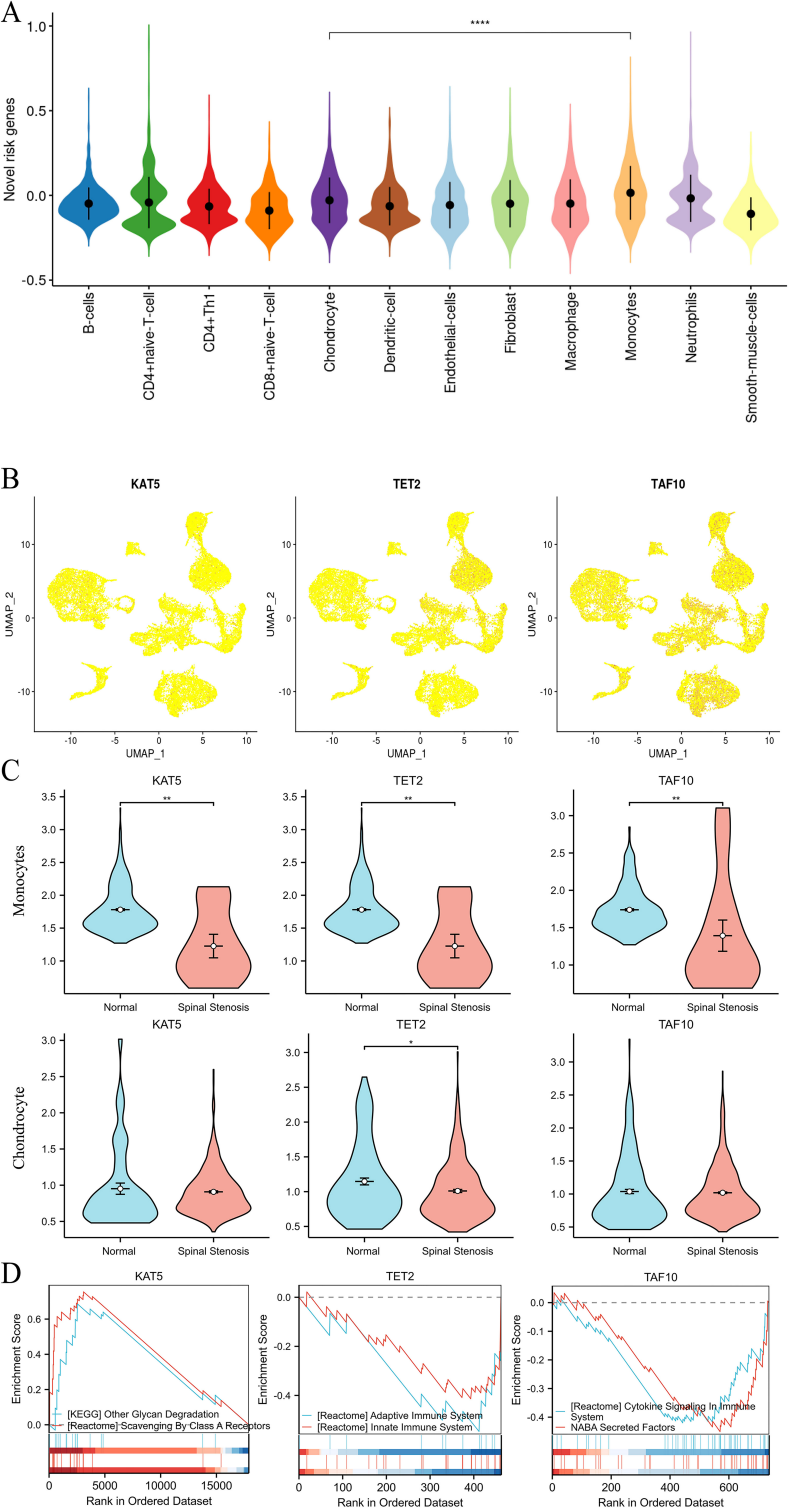
The distribution and pathway enrichment of three target genes. **(A)** The novel risk genes in different cell types. **(B)** The three target genes expression level in different cell types. **(C)** The three target genes relative distribution in spine tissues. **(D)** The GSEA results of KAT5, TET2, and TAF 10. *P < 0.05, **P < 0.01, ****P < 0.001.

After identifying alterations in cell type composition associated with spinal stenosis, we furtherly investigated the expression levels of three target genes in actual spinal stenosis tissue samples. The expression patterns of three target genes in 12 cell types were shown in Figure 4A. Among them, monocytes exhibited the highest expression levels. Notably, there were statistically significant differences in the expression of the three target genes between chondrocytes and monocytes.

**FIGURE 5:**
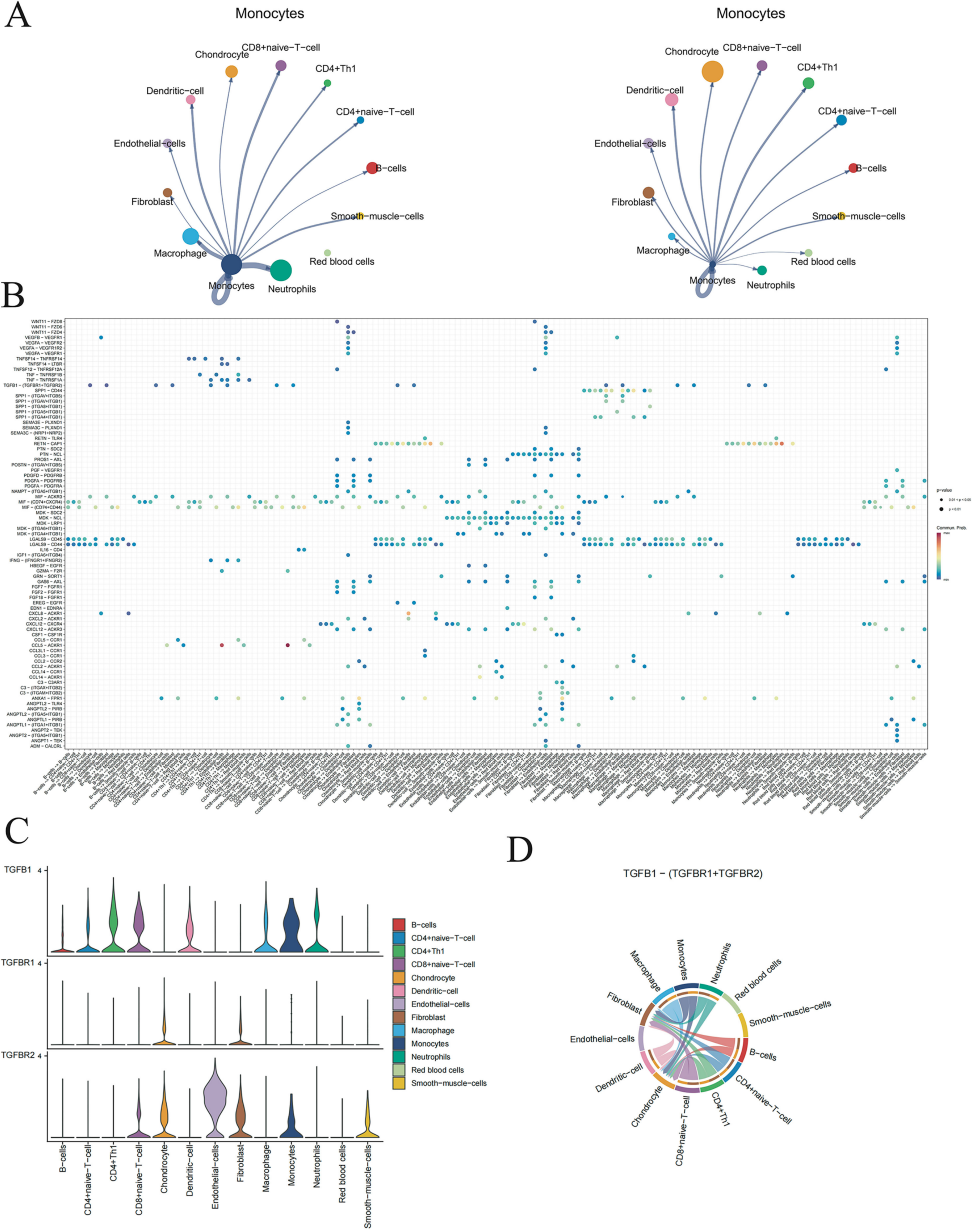
The interaction between different cell types. **(A)** The weight of interaction between different cell types in normal tissue and spinal stenosis tissue. **(B)** Each interaction signal’s credibility between different cell types. **(C)** Expression level of TGF-β1, TGF-βR1, and TGF-β2 in each cell type. **(D)** The weight of TGF-B1–(TGF-βR1 + TGF-βR2) in the interaction between different cell types.

The spatial distribution of KAT5, TET2, and TAF10 within spinal stenosis tissues was illustrated in Figure 5B. Combine with Figures 4A, B, we found that both TET2 and TAF10 are highly expressed in monocytes and KAT5 shows high expression in chondrocytes which indicated that the change of those three target genes may influence spinal stenosis by altering the chondrocyte and monocytes, even their interactions (Figure 4C).

The single gene pathway enrichment of KAT5, TET2, and TAF10 based on GSEA was subsequently conducted (Figure 4D). KAT5 was enriched in glycan degradation, TET2 was enriched in adaptive immune system and TAF10 was enriched in cytokine signaling in immune system. Furthermore, the weight of interaction between different cell types in both normal tissue and spinal stenosis tissue was explored (Figure 5A). All interaction signals between different cell types were presented in Figure 5B and one pathway stand out as it has the lowest *p*-value in many interactions between different cell types which is TGF-B1-(TGF-BR1 + TGF-BR2). The expression level of TGF-B1, TGF-BR1, and TGF-BR2 in each cell type was shown in Figure 5C and the weight of TGF-B1-(TGF-BR1 + TGF-BR2) in the interaction of different cell types was visualized in Figure 5D.

To identify active pharmacological agents targeting the genes of interest, we also performed high-throughput virtual screening of the corresponding target proteins using the FDA-approved drug library. Subsequently, based on docking scores, binding interactions, visual analysis, and elimination of redundant molecules, we selected the candidate drug molecules demonstrating high affinity for each target and manually screened them one by one according to drug library and relevant literature.

**FIGURE 6:**
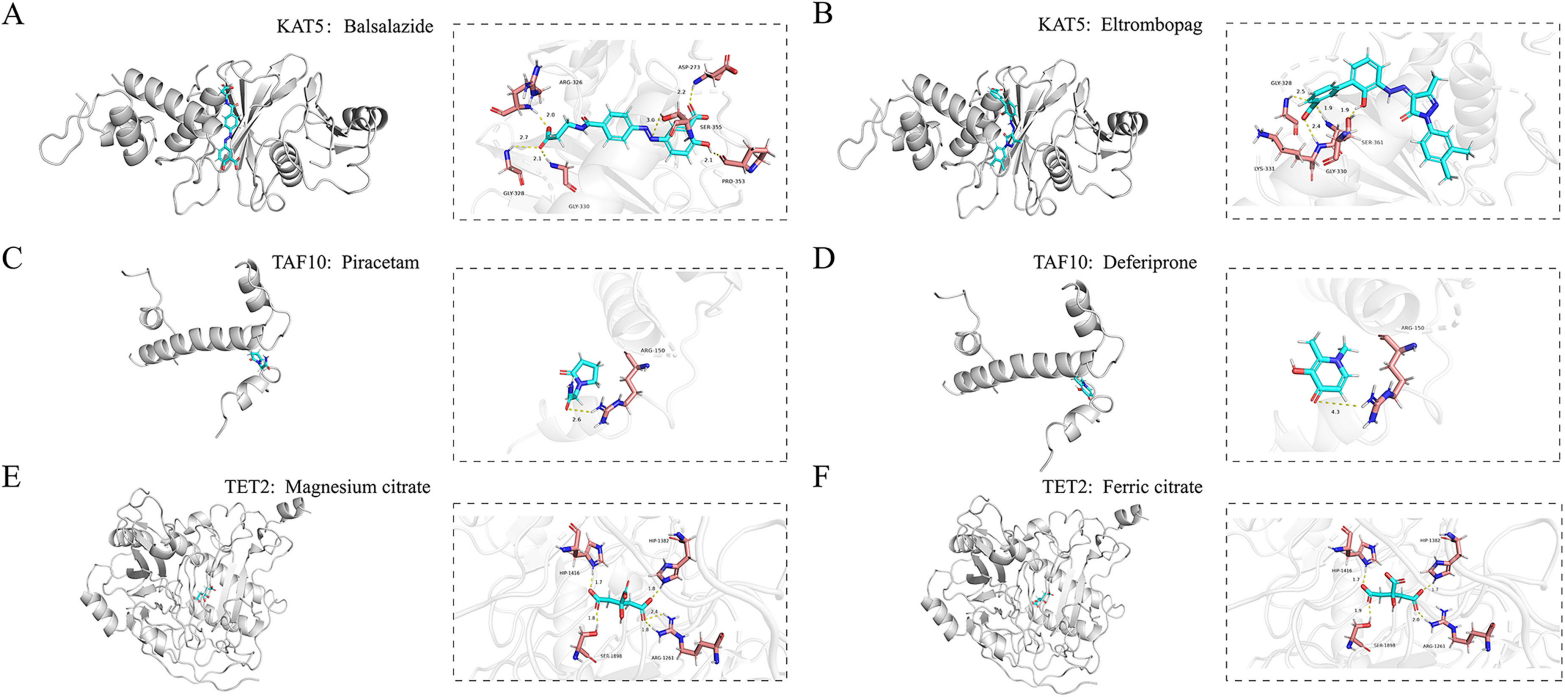
Binding mode of proteins and ligands based on molecular docking. **(A)** Binding mode of KAT5 with Balsalazide. **(B)** Binding mode of KAT5 with Eltrombopag. **(C)** Binding mode of TAF10 with Piracetam. **(D)** Binding mode of TAF10 with Deferiprone. **(E)** Binding mode of TET2 with Magnesium citrate. **(F)** Binding mode of TET2 with Ferric citrate.

Subsequently, we analyzed the three-dimensional binding modes and molecular interactions between the top-ranked compound and each target protein, as depicted in Figure 6. Each target was matched with two available chemical drugs and values of docking score <−5 kcaL/moL are generally considered indicatives of strong affinity.

The two compounds interacted with KAT5 are Balsalazide and Eltrombopag. The docking score of the calculated binding affinity between Balsalazide and KAT5 is −8.87 (Figure 6A) and the docking score between Eltrombopag and KAT5 is −8.845 (Figure 6B) which indicated strong robust binding energy. Piracetam, Deferiprone were picked out as therapeutic drugs targeting TAF10 (Figures 6C,D). The docking scores of binding affinities for them were calculated as −5.097 and −5.062, demonstrating favorable binding energy consistent with biologically relevant interactions. Magnesium citrate, Ferric citrate are two selected targeting TET2 drugs (Figures 6E,F). The docking scores for Magnesium citrate, Ferric citrate were, respectively, determined to be −5.097 and −5.029, further corroborating their binding capacity.

**FIGURE 7:**
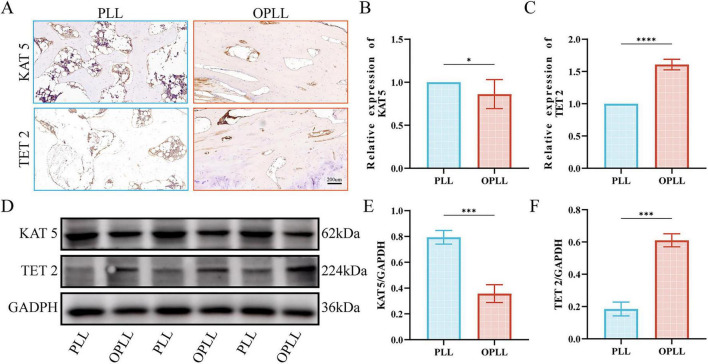
The validation of target proteins in real world human samples. **(A)** Immunohistochemical staining of target proteins on both OPLL tissues and PLL tissues. **(B)** The relative expression level of KAT5 in OPLL tissues compared with PLL tissues. **(C)** The relative expression level of TET2 in OPLL tissues compared with PLL tissues. **(D)** The Western Blot of target proteins in primary cells from posterior longitudinal ligament tissues of both OPLL and PLL. **(E)** The expression level of KAT5 in primary cells of posterior longitudinal ligament tissues from both OPLL and PLL. **(F)** The expression level of TET2 in primary cells of posterior longitudinal ligament tissues from both OPLL and PLL. Three independent experiments were performed. The statistical analysis was performed by One-Way ANOVA, and *p* < 0.05 was considered significance. *indicated *p* < 0.05, ***indicated *p* < 0.001 and *⁣*⁣**indicated *p* < 0.0001.

Finally, as the antibody of TAF10 was unavailable, we examined the other two target proteins, KAT5 and TET2, in both tissues and primary cells. The Figure 7A shows that the KAT5 and TET2 staining results of both PLL and OPLL tissues and the relative expression level of those two proteins was visualized in Figures 7B,C. The expression level of KAT5 in OPLL tissues is significantly lower than PLL tissues while TET2 is significantly higher in OPLL tissues than in PLL tissues which is consistent with our former analysis results. We also performed WB to examine their expression levels in primary cell lines and used GADPH as internal control (Figure 7D). The results also proved that the expression level of KAT5 in OPLL cell lines is significantly lower than in PLL cell lines (Figure 7E) and the expression level of TET also shows the opposite trend (Figure 7F). As OPLL is one of the main etiologies of spinal stenosis, both IHC results and WB results could validate that KAT5 may have a negative correlation with spinal stenosis and TET10 may have a positive correlation with spinal stenosis.

## Discussion

Spinal stenosis is a common degenerative disorder that severely affects quality of life and poses a major socioeconomic burden. Given the lack of effective pharmacological treatments and reliance on surgery, identifying pathogenic genes and mechanisms is urgently needed to advance targeted therapies. In this study, we employed summary data-based Mendelian randomization (SMR) methods using cis-eQTL data from the eQTLGen Consortium and spinal stenosis data from the FINNGEN database, screening from 19,960 genes and identifying 45 potential target genes. Subsequent gene-gene interaction network analysis (GGINA), refined these to 15 hub genes, and further colocalization analysis identified three key candidate genes: KAT5, TET2, and TAF10. To elucidate their biological roles, a secondary analysis of single-cell transcriptomic data from publicly available spinal stenosis datasets was conducted, revealing their enrichment in monocytes and chondrocytes, two cell types with crucial cross-talk mechanisms that modulate cartilage degeneration and inflammatory microenvironment dynamics. Specifically, the TGF-β signaling pathway emerged as a key regulator in this process. Finally, we employed molecular docking techniques to screen the FDA-approved drug database, identifying six promising compounds that may modulate the activity of these target genes. These findings provide a foundation for further exploration of the molecular mechanisms and potential pharmacological interventions for spinal stenosis.

To identify potential pathogenic and therapeutic target genes, we integrated three complementary approaches. Summary data-based Mendelian randomization (SMR), a statistical method combining GWAS summary statistics with eQTL data, enables the identification of causal genes associated with complex diseases, facilitating drug repurposing and gene expression-based therapeutic strategies (Huang et al., 2021). This approach has been widely applied in cardiovascular and autoimmune diseases and so on (Bai et al., 2024; Sun Z. et al., 2024; Zou and Yang, 2024). To further refine candidate genes, we incorporated gene-gene interaction network analysis (GGINA), a systems biology tool that maps gene interactions to identify key hub genes (Ouyang et al., 2020), narrowing our selection to 15 highly relevant targets. While HEIDI testing and FDR correction within SMR helped mitigate pleiotropic and heterogeneous effects, additional validation was necessary. We therefore employed colocalization analysis, a bioinformatics method that assesses whether GWAS and eQTL signals share the same causal variant, preventing false positives due to independent associations (Wu S. et al., 2024). As SMR alone cannot fully distinguish causal from independent associations, combining colocalization analysis improves confidence that identified genes are truly causal and biologically relevant to spinal stenosis. This multi-step approach is widely used to strengthen genetic studies of complex diseases (Sun X. et al., 2024).

Spinal stenosis is a degenerative disorder primarily caused by disc herniation, osteophyte formation, ligamentum flavum hypertrophy, and facet joint hypertrophy. Among the key pathogenic factors, degenerative lesions such as intervertebral disc degeneration and hypertrophy of peripheral ligaments and facet joints are considered primary contributors to disease progression (Lee et al., 2020). KAT5 (also known as Tip60), a lysine acetyltransferase (HAT), plays a crucial role in various cellular processes, including histone modification, transcription regulation, apoptosis, and DNA damage repair (Shvedunova and Akhtar, 2022). Our SMR analysis revealed decreased KAT5 expression, which may contribute to disease pathology, as KAT5 is essential for osteoblast and chondrocyte function, maintaining bone and cartilage health (Chandra and Rajawat, 2021). Reduced KAT5 activity can accelerate the progression of bone degenerative conditions, consistent with the pathophysiology of spinal stenosis (Greenbaum et al., 2022). Presumably, diminished KAT5 activity may exacerbate degenerative lesions, further promoting spinal stenosis. Additionally, these degenerative changes are often accompanied by increased local inflammation, characterized by elevated levels of cytokines such as TNF-α, IL-1β, and IL-6, which are known to enhance pain and contribute to further neural inflammation (Shvedunova and Akhtar, 2022). This inflammation exacerbates neural root compression and worsens pain and neurological dysfunction, hallmark symptoms of spinal stenosis. Furthermore, diseases such as rheumatoid arthritis (RA) and ankylosing spondylitis (AS), which are characterized by inflammation and altered immune responses, also lead to conditions like facet joint inflammation and ligament calcification (Sabri et al., 2019). Xu et al. (2024) has reported KAT5 expression in the peripheral blood of patients with rheumatoid arthritis and dry syndrome, implicating KAT5 in immune regulation. Mechanistically, KAT5 may influence Th17 cell differentiation via interaction with the transcription factor FOXP3 in ankylosing spondylitis (Xu et al., 2024. This hypothesis is supported by previous studies linking KAT5 to immune-related diseases, which may indirectly contribute to spinal stenosis progression via enhanced inflammation. Ligamentum flavum hypertrophy, calcification, and posterior longitudinal ligament ossification are major contributors to spinal stenosis. KAT5 regulates TGF-β-related genes via acetylation, and TGF-β signaling promotes ECM deposition and fibrosis through the SMAD pathway, driving tissue calcification (Schulz et al., 2016). Additionally, overactivation of TGF-β/SMAD signaling in fibroblasts leads to abnormal accumulation of matrix proteins, promoting fibrosis-related diseases (Zhou et al., 2024). Therefore, it is reasonable to speculate that low KAT5 expression may influence ECM metabolism via TGF-β/SMAD signaling, promoting ligamentum flavum fibrosis and ossification of the posterior longitudinal ligament.

TET2 (Ten-Eleven Translocation 2) is an enzyme involved in DNA demethylation and plays a critical role in regulating cell differentiation, inflammatory responses, ECM metabolism, and osteoblast differentiation (Cong et al., 2021; López-Moyado et al., 2024). The upregulation of TET2 in spinal stenosis may contribute to disease progression through multiple mechanisms, including ligament ossification, intervertebral disc degeneration, and chronic inflammation. Li et al. demonstrated that osteogenic markers such as RUNX2, OCN, and ALP are significantly upregulated in the ligamentous tissue of spinal stenosis patients, suggesting a critical role of osteogenic differentiation in disease progression (Li et al., 2024). Supporting this, Kubota S et al identified TET2 as a key regulator of RUNX2 (Kubota et al., 2019), which may promote ligamentous ossification, a hallmark of OLF. In addition, given that fibrosis often precedes ossification in spinal stenosis, Sharma and Bhonde proposed that TET2 can epigenetically modulate TGF-β-related genes, which are crucial for fibroblast proliferation and differentiation (Sharma and Bhonde, 2023), meanwhile, it have been clarified that TET2 regulates the TGF-β1/SMAD axis, influencing ECM metabolism and fibrosis (De et al., 2021). These findings suggest that TET2 overexpression may drive fibrosis, leading to ligament ossification and further narrowing of the spinal canal. Except for the ligament ossification, intervertebral disc degeneration is another major contributor to spinal stenosis. Liang et al. reported that TET2 can regulate p53-related genes, promoting cell senescence and apoptosis, which may contribute to intervertebral disc degeneration and herniation (Liang et al., 2024; Zhang et al., 2023). These findings indicated that excessive TET2 activity could accelerate degenerative changes in spinal structures, exacerbating disease progression. Furthermore, chronic inflammation plays a crucial role in spinal stenosis by exacerbating neural compression and pain. De et al has reported that TET2 upregulation enhances pro-inflammatory cytokine expression via NF-κB signaling, contributing to persistent inflammation (Gerecke et al., 2022). TET2 may modulate inflammatory pathways that contribute to spinal stenosis progression. Collectively, these findings suggest that TET2 overexpression may promote spinal stenosis through RUNX2-mediated ossification, TGF-β-driven fibrosis, ECM degradation, and NF-κB inflammatory signaling, making it a potential therapeutic target for disease intervention.

Another target gene identified in this study was TAF10. TAF10 (TATA-box binding protein Associated Factor 10) is a key component of the TFIID and SAGA complexes, playing a central role in RNA Pol II-mediated transcription initiation (Papadopoulos et al., 2015). Although relatively little research has been conducted on the TAF family, previous studies have shown that TAF10 plays a critical role in stem cell differentiation, particularly during neuronal differentiation and muscle cell differentiation (Luna-Arias and Castro-Muñozledo, 2024). TAF10 regulates gene transcription to drive cell differentiation, and may influence joint cell fate, such as ligamentous stem cells, thereby contributing to ligamentum flavum hypertrophy or posterior longitudinal ligament ossification in spinal stenosis.

Single-cell RNA sequencing (scRNA-seq) is an advanced high-throughput technology that enables the examination of gene expression at the single-cell level, facilitating the study of cellular heterogeneity, cellular states, and dynamic cellular changes (Ma et al., 2023; Papalexi and Satija, 2018). This technique has become a cornerstone in various fields such as oncology, immunology, neuroscience, and developmental biology (Jovic et al., 2022). Recent scRNA-seq studies have highlighted the significant roles of distinct cell populations in degenerative diseases, including spinal stenosis (Kambouris et al., 2014; Maffulli et al., 2013). For example, Tang et al. (2024) employed scRNA-seq to investigate cell populations associated with spinal ligament degeneration, focusing on CRTAC1+ cells and SPP1+ macrophages, which were identified as key players in the progression of spinal stenosis. Similarly, scRNA-seq has been used to examine the roles of immune cells and nucleus pulposus cells in intervertebral disc degeneration, a leading cause of spinal stenosis (Ling et al., 2022). These studies underscore the utility of scRNA-seq in uncovering cellular diversity and molecular mechanisms, providing critical insights into spinal stenosis pathophysiology and identifying potential therapeutic targets and biomarkers (Kambouris et al., 2012). In this study, we re-analyzed publicly available scRNA-seq data for spinal stenosis and integrated the results with previous Mendelian randomization findings that identified three target genes. Re-clustering revealed significant monocyte-chondrocyte cross-talk, suggesting their potential role in disease progression.

Chondrocytes, the principal cells of cartilage, are crucial for matrix synthesis and maintenance, tissue repair, and immune response regulation (Akkiraju and Nohe, 2015). Dysregulation of chondrocyte function, particularly in degenerative cartilage diseases, can lead to cartilage damage and degenerative changes (Terkawi et al., 2022). Several scRNA-seq studies have identified unique subsets of chondrocytes and emphasized their involvement in spinal diseases, especially intervertebral disc degeneration. For instance, Zang et al. reported the identification of distinct chondrocyte subpopulations in human intervertebral discs and highlighted the role of ferroptosis in disc degeneration (Zhang et al., 2021), further implicating chondrocytes in spinal stenosis. In addition, Yabe et al. (2015) demonstrated that chondrocytes contribute to the pathogenesis of lumbar spinal canal stenosis by participating in ligamentum flavum hypertrophy and fibrosis. Their research confirmed that chondrogenesis and fibrosis are pivotal processes in the pathological changes of the ligamentum flavum, ultimately leading to spinal deformities (Wu K. et al., 2024). These findings underscore the essential role of chondrocytes in spinal stenosis progression, particularly through cartilage degradation and fibrosis in ligamentous tissues.

Monocytes are central to immune responses and inflammation (Geissmann et al., 2010), interacting with various cell types, including chondrocytes, to modulate tissue homeostasis and pathological processes. Ou et al. (2022) explored the MCP-1/CCR2 axis in human nucleus pulposus mesenchymal stem cells and demonstrated that monocytes, via the CCR2 receptor, inhibit chondrogenic differentiation, impairing cartilage repair. This interaction suggests that monocytes and chondrocytes engage in cross-talk that may influence cartilage degradation and contribute to spinal stenosis progression. In the context of our analysis, we found that the communication between monocytes and chondrocytes is mediated, at least in part, through the TGF-β signaling pathway.

TGF-β is a multifaceted cytokine essential for various biological processes, including cell proliferation, differentiation, tissue repair, and immune regulation (Dahmani and Delisle, 2018). TGF-β signaling is crucial in regulating fibrosis, hypertrophy, and extracellular matrix (ECM) remodeling (Caja et al., 2018). Several studies have highlighted the involvement of TGF-β signaling in spinal stenosis, particularly in ligamentum flavum hypertrophy. For instance, Mario et al. found a close association between TGF-β expression and ligamentum flavum hypertrophy in spinal stenosis, suggesting its central role in the inflammatory response (Löhr et al., 2011). Similarly, Wang et al. (2021) demonstrated that the TGF-β/Smad signaling pathway significantly contributes to ligamentum flavum hypertrophy, providing deeper molecular insights into the process. Collectively, these studies indicate that TGF-β signaling is a key driver of ligamentum flavum fibrosis and hypertrophy, central to spinal stenosis pathogenesis. Our findings further suggest that monocyte-chondrocyte interactions, particularly via the TGF-β pathway, play a crucial role in disease progression. Targeting this pathway or modulating monocyte and chondrocyte function may provide a promising therapeutic approach. However, further research is needed to validate these findings and explore potential interventions.

Molecular docking is a computational technique that predicts interactions between small molecules and target proteins, aiding in drug repurposing and therapeutic development (Pinzi and Rastelli, 2019). This study screened FDA-approved drugs against KAT5, TET2, and TAF10, genes implicated in spinal stenosis. For instance, Balsalazide, a treatment for ulcerative colitis, exerts anti-inflammatory effects through 5-aminosalicylic acid (5-ASA, Mesalamine), which inhibits NF-κB signaling and reduces pro-inflammatory cytokines (Dhillon and Singh, 2020; Di Mari et al., 2007). Given KAT5’s role in NF-κB-dependent inflammation, its strong binding with Balsalazide suggests potential for suppressing inflammation in spinal stenosis. Similarly, Piracetam, a nootropic drug that enhances synaptic plasticity and neurotransmission (Nomura and Nishizaki, 2000), indicating its potential in neuroprotection and nerve recovery, key in spinal stenosis treatment. Additionally, rat models of lumbar spinal stenosis have shown that iron overload-induced oxidative stress exacerbates spinal tissue damage (Hong et al., 2022), with iron accumulation primarily occurring in M1 pro-inflammatory macrophages, sustaining an inflammatory environment (Kim et al., 2022). Given the link between iron overload, oxidative stress, and inflammation, the iron chelator Deferiprone, may help reduce iron accumulation and mitigate inflammation, potentially slowing spinal stenosis progression (Bahir et al., 2024). These findings underscore the potential of molecular docking in identifying new applications for existing drugs, yet it should be noted that molecular docking provides only a static prediction of ligand–protein interactions. Docking scores do not account for protein conformational dynamics, cellular context, pharmacokinetics, or pharmacodynamics. Therefore, the identified compounds should be considered candidate molecules requiring further biochemical and *in vivo* validation rather than confirmed therapeutic agents.

This study offers valuable insights, however, several limitations should be acknowledged. First, the data were exclusively derived from the FINNGEN biobank, where the majority of participants are of European ancestry, potentially limiting the generalizability of our findings to other ethnic groups. Future studies should incorporate more diverse populations to enhance the applicability of these results. Second, the GWAS dataset lacked detailed subgroup information for spinal stenosis classification. Given the disease’s heterogeneity, a more refined stratification based on etiology—such as intervertebral disc degeneration, ligamentum flavum hypertrophy, or ossification—is necessary to better understand genetic predispositions and therapeutic responses (Hareni et al., 2024). Third, while single-cell sequencing is a powerful tool, it is inherently constrained by the limited number of transcripts captured per cell, which may hinder the detection of lowly expressed genes and obscure the full extent of cellular heterogeneity (Artells et al., 2016). Advances in single-cell multi-omics and spatial transcriptomics could help overcome these challenges by providing more comprehensive molecular profiles. Fourth, although this study identified three key target genes and six potential therapeutic compounds, their expression patterns *in vivo* and *in vitro*, as well as their therapeutic efficacy in spinal stenosis, require further validation. Moving forward, integrating larger and more diverse cohorts, leveraging multi-omics approaches, and conducting functional validation studies will be crucial for translating these genomic discoveries into effective therapeutic strategies for spinal stenosis.

Building upon previous evidence, our findings suggest that spinal stenosis progression is driven by a complex interplay of genetic, cellular, and molecular factors centered on the TGF-β signaling pathway. The three key genes identified—KAT5, TET2, and TAF10—appear to modulate fibrotic and ossific changes through regulation of extracellular matrix deposition, osteogenic differentiation, and inflammatory responses. Specifically, decreased KAT5 expression may impair osteoblast and chondrocyte function while promoting inflammation via NF-κB signaling, consistent with its role in immune-mediated diseases. TET2 upregulation likely exacerbates fibrosis and ossification by epigenetically regulating TGF-β/SMAD and RUNX2 pathways and enhancing pro-inflammatory cytokine expression. TAF10, as a transcriptional regulator, may influence the differentiation of ligamentous stem cells, contributing to ligament hypertrophy and ossification. Single-cell transcriptomics highlights crucial cross-talk between monocytes and chondrocytes mediated by these pathways, which orchestrates cartilage degradation and inflammatory microenvironment dynamics. Our molecular docking results identify several FDA-approved drugs that potentially target these genes and their associated signaling cascades, opening opportunities for therapeutic repurposing. Collectively, this integrative model unifies genetic predisposition, cell-cell interactions, and molecular signaling in spinal stenosis pathogenesis and provides a rational basis for future experimental validation and targeted intervention.

## Conclusion

This study integrates Mendelian randomization, single-cell transcriptomics, and molecular docking to reveal key genes (KAT5, TET2, TAF10) driving spinal stenosis via TGF-β-related mechanisms. Six FDA-approved drugs showed strong binding potential, suggesting repurposing opportunities. These findings advance molecular insights and support precision therapeutics for spinal stenosis, though further experimental validation is needed.

## Data Availability

The datasets presented in this study can be found in online repositories. The names of the repository/repositories and accession number(s) can be found in the article/supplementary material.

## Footnotes

1
https://www.eqtlgen.org/phase1.html
